# Structural and functional influences of coagulation factor XIII subunit B heterozygous missense mutants

**DOI:** 10.1002/mgg3.138

**Published:** 2015-04-10

**Authors:** Anne Thomas, Arijit Biswas, Vytautas Ivaskevicius, Johannes Oldenburg

**Affiliations:** Institute of Experimental Haematology and Transfusion Medicine, University Clinic Bonn53127, Bonn, Germany

**Keywords:** Confocal microscopy, FXIIIB subunit, missense mutation, molecular dynamic simulation, secretion defects

## Abstract

The coagulation factor XIII(FXIII) is a plasma circulating heterotetrameric protransglutaminase that acts at the end of the coagulation cascade by covalently cross-linking preformed fibrin clots (to themselves and to fibrinolytic inhibitors) in order to stabilize them against fibrinolysis. It circulates in the plasma as a heterotetramer composed of two homomeric catalytic Factor XIIIA_2_ (FXIIIA_2_) and two homomeric protective/carrier Factor XIIIB_2_ subunit (FXIIIB_2_). Congenital deficiency of FXIII is of two types: severe homozygous/compound heterozygous FXIII deficiency which results in severe bleeding symptoms and mild heterozygous FXIII deficiency which is associated with mild bleeding (only upon trauma) or an asymptomatic phenotype. Defects in the *F13B* gene (Factor XIIIB subunit) occur more frequently in mild FXIII deficiency patients than in severe FXIII deficiency. We had recently reported secretion-related defects for seven previously reported *F13B* missense mutations. In the present study we further analyze the underlying molecular pathological mechanisms as well as the heterozygous expression phenotype for these mutations using a combination of in vitro heterologous expression (in *HEK293T* cells) and confocal microscopy. In combination with the *in vitro* work we have also performed an *in silico* solvated molecular dynamic simulation study on previously reported FXIIIB subunit sushi domain homology models in order to predict the putative structure-functional impact of these mutations. We were able to categorize the mutations into the following functional groups that: (1) affect antigenic stability as well as binding to FXIIIA subunit, that is, *Cys5Arg*, *Cys316Phe*, and *Pro428Ser* (2) affect binding to FXIIIA subunit with little or no influence on antigenic stability, that is, *Ile81Asn* and *Val401Gln* c) influence neither aspects and are most likely causality linked polymorphisms or functional polymorphisms, that is, *Leu116Phe* and *Val217Ile*. The *Cys5Arg* mutation was the only mutation to show a direct secretion-based defect since the mutated protein was observed to accumulate in the endoplasmic reticulum.

## Introduction

Factor XIII deficiency is a rare bleeding disorder that results from the deficiency of coagulation FXIII, a hetero-tetrameric protransglutaminase molecule that functions by cross-linking preformed fibrin clots to provide them mechanical stability and resistance to fibrinolysis (Lorand et al. 1980). The plasma circulating FXIII is composed of two catalytic FXIIIA_2_ subunits and two protective FXIIIB_2_ subunits. While the homozygous inherited form of this deficiency caused by *FXIIIA* (OMIM #613225) or *FXIIIB* (OMIM #613235) gene mutations is rare (1 in 4–6 million), the milder heterozygous form is more frequent (Biswas et al. 2011; Biswas et al. 2014a,b). Only recently, focus has shifted to the mild/heterozygous form of this deficiency that is associated with mild or even an asymptomatic phenotype (unless the affected individual is exposed to some kind of a trauma, for example, perioperative settings, accident etc.). Four publications from our group in the past 5 years have shown that inherited mild or heterozygous deficiency does have clinical relevance (Biswas et al. 2010; Ivaskevicius et al. 2010a,b; Biswas et al. 2014a,b). One key observation from these articles is that the FXIIIB subunit mutations, which are rarely reported in the severe homozygous form of FXIII deficiency, occur at almost equal proportion when compared with the frequency of FXIIIA subunit mutations in mild heterozygous FXIII deficiency (Ivaskevicius et al. 2010b). It is secreted as a homodimer into the plasma where it associates with the FXIIIA_2_ subunit to form the FXIIIA_2_B_2_ heterotetramer (Radek et al. 1993). It is secreted in excess of the FXIIIA_2_ subunit in the plasma. Therefore, there is always free FXIIIB subunit aside from the complexed one circulating in the plasma. Despite early achievements investigating FXIIIB secondary structural elements and structural domains (Ichinose et al. 1986), progress on structure/function studies of this noncatalytic subunit have been slow and there are no high-resolution x-ray-based crystal/NMR structures for FXIIIB_2_ dimers, or for the FXIIIA_2_-bound conformation in FXIIIA_2_B_2_ tetramers. Based on gel filtration chromatographic data, the FXIIIB subunit appears to form a physiological dimer (Souri et al. 2008). High sequence homologies with proteins from the complement system suggest that the monomeric B subunit is composed of ten Sushi domains, each comprising ∼60 amino acid residues and also designated in the literature as complement control protein (CCP) modules, or as short consensus repeats (SCR) (Ichinose et al. 1986). Knowledge of the structural interfaces between individual FXIIIB_2_ subunit dimers as well as for the intermolecular interfaces of the FXIIIA_2_B_2_ heterotetramer has only recently started to emerge (Katona et al. 2014). The sushi domain folds into a small and compact hydrophobic core enveloped by six *β*-strands which are stabilized by two disulfide bridges on either end of this domain. All sushi structures share the relative structural orientation of the *β*-2 and *β*-4 strands. The other strands vary in topology relative to this central conserved core especially at the interdomain interfaces (Gaboriaud et al. 2000). There are more than 25 high-resolution structures for this highly conserved domain type and additional Sushi domain-containing protein (Soares et al. 2005). In a recent article we have reported homology-based models for all ten sushi domains of the FXIIIB subunit based on the templates of complement factor H (CFH) sushi domains (Biswas et al. 2013). We have so far reported 12 unique mutations in the FXIIIB_2_ subunit from patients with heterozygous (mild) FXIII deficiency of which seven were missense mutations. In an earlier article using heterologous in vitro expression we had shown that almost all reported FXIIIB subunit missense mutations showed differences in secretion rate, with the highest impact being observed for two mutations affecting structural disulfide bonds and one mutation involving a Proline428 residue (Biswas et al. 2013). However, a few questions remained unanswered from this article which we have now attempted to answer in our present study. These questions are: (1) Do the differences in secretion rates correspond to a genuine secretion defect, that is, is there intraorganellar accumulation/aggregation or are they simply a reflection of the influence that the mutations have on the rate of biosynthesis and folding? (2) Does the in vitro expression data of the *F13B* mutations correlate with the heterozygous patient phenotype? (3) Which mutations are most likely to affect binding to the FXIIIA_2_ subunit without compromising the antigenic stability of the FXIIIB_2_ subunit? (4) What are the likely structure-functional correlations for these mutations? In our present study we have used a combination of heterologous *in vitro* expression, confocal microscopy and molecular dynamic simulation (MD) on homology-based models to address these questions.

## Material and Methods

### Cell culture, transfection, and protein expression and quantification

Site-directed mutagenesis for the seven FXIIIB missense mutations were performed on a mammalian expression pEZ-MO1-*FXIIIB* vector using the Gene Tailor site-directed mutagenesis kit (Life Technologies, Darmstadt, Germany). The seven FXIIIB mutant variants [pEZ-MO1-FXIIIB-p.Cys5Arg (*F13Bc.73T>C*), pEZ-M01-FXIIIB-p.Cys316Phe (*F13Bc.1007G>T*), MO1-FXIIIB-p.Ile81Asn (*F13B*c.302T>A), MO1-FXIIIB-p.Leu116Phe (*F13B*c.406C>T), MO1-FXIIIB-p.Val217Ile (*F13Bc*.709G>A), MO1-FXIIIB-p.Val401Glu (*F13B*c.1262T>A), MO1-FXIIIB-p.Pro428Ser (*F13B*c.1342C>T)] were expressed in *HEK 293T* cells (DSMZ [Institute of DSMZ-German Collection of Microorganisms and Cell Cultures, Germany]) in homozygous and heterozygous forms, that is, mutations or the wild type was expressed alone (homozygous), mutations were cotransfected with the wild type (heterozygous). Culturing of the cells was done in 10 cm dishes with Dulbecco′s modified Eagles medium (DMEM; Life Technologies) supplemented with 10% fetal bovine serum (FBS; Life Technologies), 1% penicillin-streptomycin (Life Technologies) and 0.1% Fungizone (Life Technologies) at 37% in 5% CO_2_. For transfection 2.7 × 10^6^ million cells were seeded out into 6-well plates with DMEM (with FBS) without supplements and transfected with wild-type and mutated DNA using Lipofectamine 2000 reagent (Life Technologies). Co-transfection of the variants was done at a 4:0.5 ratio with a pCMV-LacZ Vector (Clontech, Saint-Germain-en-Lay, France) containing the *LacZ* gene for normalizing the antigen values. All samples (supernatant and lysed cells) were collected 36 h after media change. The extracellular medium was centrifuged at 14,000*g* to remove cell debris and the cells were washed with phosphate-buffered saline (PBS) and lysed by incubation with 260 *μ*L nondenaturing lysis buffer “native M-PER Mammalian Protein Extraction Reagent buffer” (Life Technologies, Darmstadt, Germany) containing 25 mmol/L bicine pH 7.6 for 10 min incubation and centrifuged at 14,000*g* for 5 min at 4°C. Both extracellular medium and cell lysates were stored at −80°C for later analysis. Each transfection set was performed in triplicate and repeated a minimum of three times(total *n* > 9). Total FXIIIB_2_ and FXIIIA_2_B_2_ antigen levels were evaluated for the expression supernatants using ELISA kits (Technoclone, Vienna, Austria).

### Confocal microscopy

For confocal microscopy 270000 *HEK293T* cells were seeded on 12-mm Poly-d-Lysine coated coverslips (BD Biosciences, Heidelberg, Germany) in 24-well cell culture plates. The cells were transiently transfected with the Wild-type pEZ-MO1-FXIIIB and the seven FXIIIB mutant variants using Lipofectamine 2000 transfection reagent (Life Technologies) following the recommendations of the manufacturer. After 24 h cells were washed and fixed with 4% paraformaldehyde in PBS for 10 min and at room temperature. After washing with PBS and blocking (90% PBS azide [0.1% sodium azide], 10% FBS, 0.1% Triton-X100) incubation with 2.5 *μ*g/mL primary antibody diluted in PBS azide (1% FBS, 0.1% Triton-X100) against the FXIIIB subunit (mouse monoclonal IgG) and the cell compartments ER (IgG rabbit polyclonal anti-calnexin; Abcam, Cambridge, England) and Golgi (rabbit anti-TGn46; Sigma, Hamburg, Germany) was done. Signal detection was performed using an IgG Alexa Fluor 488 IgG goat anti-mouse (Life technologies) against the FXIIIB subunit and an IgG goat anti-rabbit Alexa Flour 594 (Life technologies) against the ER and Golgi compartment. After washing the treated cells and Dapi (Life technologies) staining for nucleus visualization the samples were embedded with VectaShield mounting medium (Vector Laboratories, Peterborough, United Kingdom) and stored at 4°C for visual imaging with the Olympus Fluo View FV 1000 confocal microscope, Hamburg, Germany.

### Image analysis

Colocalization analysis was performed using plugins embedded in the image visualization and analysis software ImageJ 1.43m (Schneider et al. 2012). Analysis was performed on a similar sized symmetrical region of interest (ROI) selected for each dye. Background levels were subtracted from each ROI before calculating the degree of colocalization (to a range of one standard deviation). Each colored image was split into the respective RGB (red, green, blue) channels. The comparative degree of colocalization for the wild-type and mutant variants was calculated as mean Pearson’s and Mander′s R coefficients on the red and green channels using the embedded colocalization analysis plugin at default settings. The colocalization highlighter plugin was used also with default setting (50% threshold values for both channels) to further visualize the co-localized pixels rendered as white. Since Pearson’s and Mander′s R coefficient showed good correlation (Mander′s R was almost consistently higher than Pearson’s correlation coefficient), therefore only Pearson’s correlation coefficient was used for comparing relative degree of colocalization between the mutant variants and wild-type (Adler and Parmryd 2010). A minimum of *n* = 10 ROI′s were evaluated for each pairwise comparison.

### Expression and reconstitution study

Constant amount of the recombinant FXIIIA_2_ subunit (Zedira, Darmstadt, Germany) (0.75 *μ*g) was added to a final volume of 200 *μ*L of transfection product (wild type as well as mutants), that is, the secreted medium for reconstituting the heterotetramer. In the event of a significant difference in the beta galactosidase reporter levels between the particular mutant and wild type the mixing volumes would have been altered to allow for differences in transfection efficiency. Since this set of expression and reconstitution showed no significant differences in beta galactosidase reporter levels the mixing volumes were not altered. Postincubation the mix was evaluated for FXIIIA_2_B_2_ heterotetrameric antigen levels. The results are normalized against total FXIIIB_2_ antigen levels previously evaluated to give a representation of the respective variant’s ability to form a heterotetramer, which are compared against the wild type. This ability we name as the comparative tetramerization potential (CTP) or potential percentage (CTPP). It is calculated by the following formula:

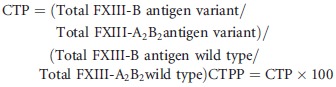


### Proteasomal inhibition

Typical 36 h transfection and collection with the wild type and different variants was performed as described above. The only difference was that after 24 h of transfection 10 mmol/L Lactacystin (to a final concentration 10 *μ*mol/L) was added to the cell medium. Therefore, the cells were incubated with 10 *μ*mol/L of Lactacystin for a period of 12 h. This analysis was done only one time in triplicates.

### Structural and sequence conservation analysis

Two different types of multiple sequence alignments were generated to evaluate the conservation of residues on which the mutations have been reported and their neighboring residues. The first alignment was generated by aligning individual FXIIIB sushi domain sequences with its near homolog CFH sushi domain amino acid sequences. Since elaborate functional information exists for the CFH sushi domains, this alignment helped us to functionally evaluate FXIIIB sushi domains with respect to functional definitions for CFH sushi domains (Perkins et al. 2012; Kopp et al. 2012). A more elaborate multiple sequence alignment was generated by first downloading sushi domain amino acid sequences of 50 mammalian proteins (Soares et al. 2005) and then aligning them with the ten FXIIIB sushi domain sequences in order to look for overall sequence conservation and identity. Alignment was done on Jalview 2.7 using MAFFT, L-insi-1 accuracy-based parameters (Waterhouse et al. 2009; Katoh and Standley et al. 2012). In addition to sequence conservation analysis, structural conservation of the FXIIIB subunit sushi domains was evaluated by submitting their homology-based models individually to the PROBIS server (http://probis.cmm.ki.si/) (Konc and Janezic 2010a,b).

### MD simulation analysis

Previously published homology-based models were used as a primer for the MD simulation study (Biswas et al. 2013). The previously generated models were first refined by a short solvated refinement simulation run of 500 ps on YASARA version 13.9.8 (Krieger et al. 2002). Individual sushi domain models were housed in a simulation cell 2 × 7.5 Å larger than the model on each axis. The simulation cell was filled with water to a density of 0.997 g/L. The YASARA YAMBER03 force field derived originally from the AMBER force field was imposed. Periodic boundary conditions were used for the simulation run. The structure with the lowest energy from the simulation run was used for further study. Each of the refined sushi domain models (wild type) on which the mutations occur were individually run for a period of 25 ns with a different force field (AMBER03) than the one used for model refining. The remaining simulation protocol was similar to the one used for model refining. The same protocol was employed for a 25-ns run for sushi domains on which the individual missense variants were introduced and optimized for the best possible rotamer (using SCRWL parameters) (Krivov et al. 2009). The simulation trajectories were analyzed for DCCM (dynamic cross-correlation matrices), C-alpha backbone RMSDs (root mean square deviation), RMSF (root mean square fluctuation), radius of gyration, surface electrostatic potential, accessible surface area and other variable components. These analyses were performed for the complete trajectory as well as for the simulation-averaged structure. The simulation variables were compared between the wild-type and mutated sushi domains. All image rendering and structural analysis (of the simulation trajectories as well as the simulation averaged structures) was done using YASARA version 13.9.8 and SWISS-PDB viewer (Guex et al. 2009). Difference in folding free energies between mutant and wild-type structures were calculated using the following web servers: Imut 2.0, SDM, McSM, DUET, ERIS (Capriotti et al. 2005; Yin et al. 2007; Worth et al. 2011; Pires et al. 2014a,b) and also with the FOLDX plug in embedded in YASARA (Van Durme et al. 2011). The simulation averaged structures for both the wild type and mutants were used as an input for these web servers as well as for FOLDX-based calculations. The PIPSA web server (http://pipsa.eml.org/pipsa/) was used to calculate the surface electrostatic potential distances between wild type and mutated sushi domain (once again the simulation averaged structures were used as an input) (Richter et al. 2008).

## Results

### Heterologous expression of FXIIIB subunit variants in homozygous and heterozygous forms

**Figure 1:**
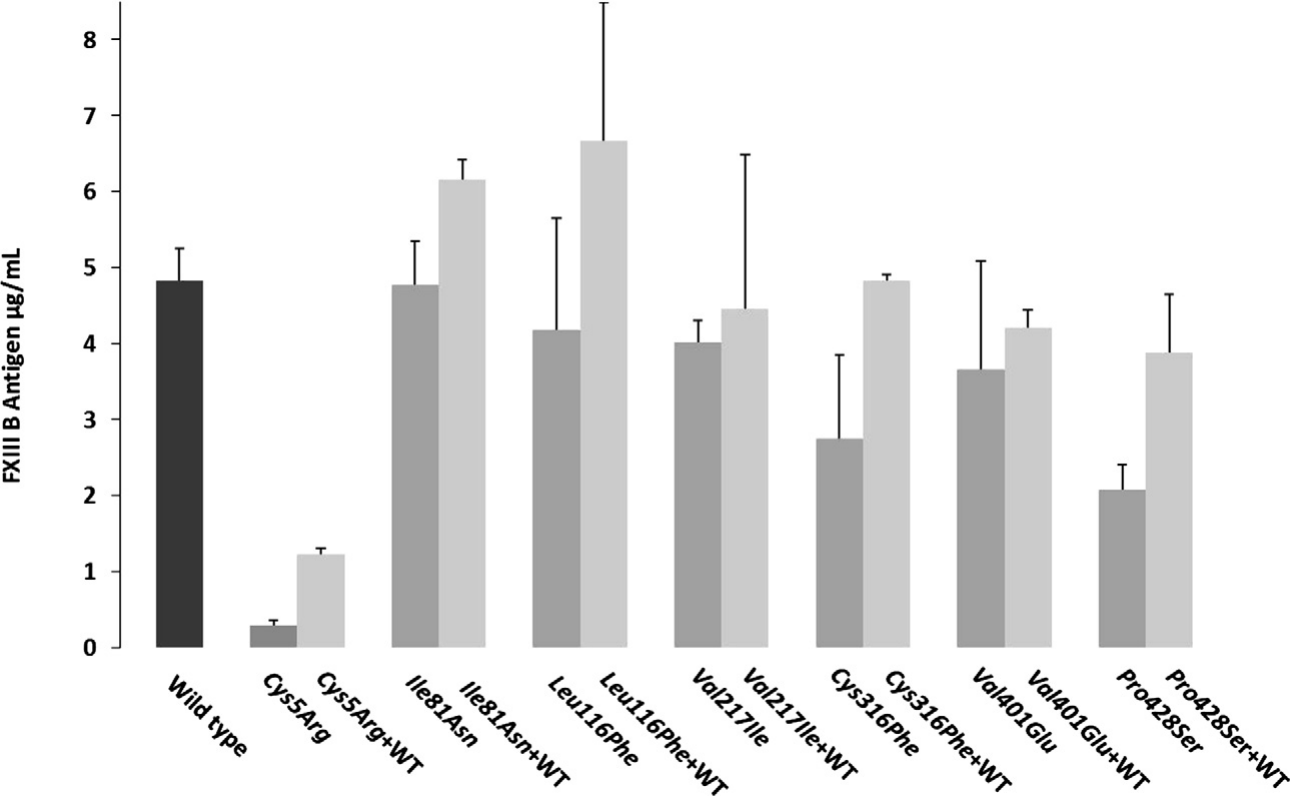
Transient homozygous/heterozygous expression of the reported missense mutations. This image represents comparative bar graph representations for the mutant and wild-type FXIIIB expression antigen values, homozygous as well as heterozygous. The leftmost black bar represents the wild type. Amongst the other bars, the dark gray represent homozygous mutant expression while the light represents the heterozygous expression (cotransfection with wild type). All values have already been normalized for transfection efficiency with beta galactosidase levels. The error bars represent the standard deviation.

The results of heterologous expression of the FXIIIB subunit wild type and mutations in homozygous form showed a pattern that was published recently (Fig.1) (Biswas et al. 2013). Three of the mutations (*Cys5Arg*, *Cys316Phe*, and *Pro428Ser*) showed quite low levels of secreted FXIIIB subunit (mean levels ranging between 0.29 and 2.74 *μ*g/mL) Another variant showed moderately reduced levels than the wild type (*Val401Glu*; mean level 3.65 *μ*g/mL), three other variants (*Ile81Asn*, *Leu116Phe*, *Val217Ile*) showed levels close to that of the wild type (4.82 *μ*g/mL). The heterozygous coexpression with the wild type for all variants afforded some degree of correction for the mutated expression phenotype. In fact in some of the variants (*Ile81Asn*, *Leu116Phe*, *Val217Ile, Cys316Phe*, and *Val401Glu*) the coexpressed phenotype FXIIIB_2_ antigen levels (4.2–6.66 *μ*g/mL) were quite similar to the wild type (Fig.1) except for *Cys5Arg* and *Pro428Ser* which reached only 1.22–3.87 *μ*g/mL FXIIIB antigen levels.

### Confocal microscopy and Image analysis for B subunit variants

**Figure 2:**
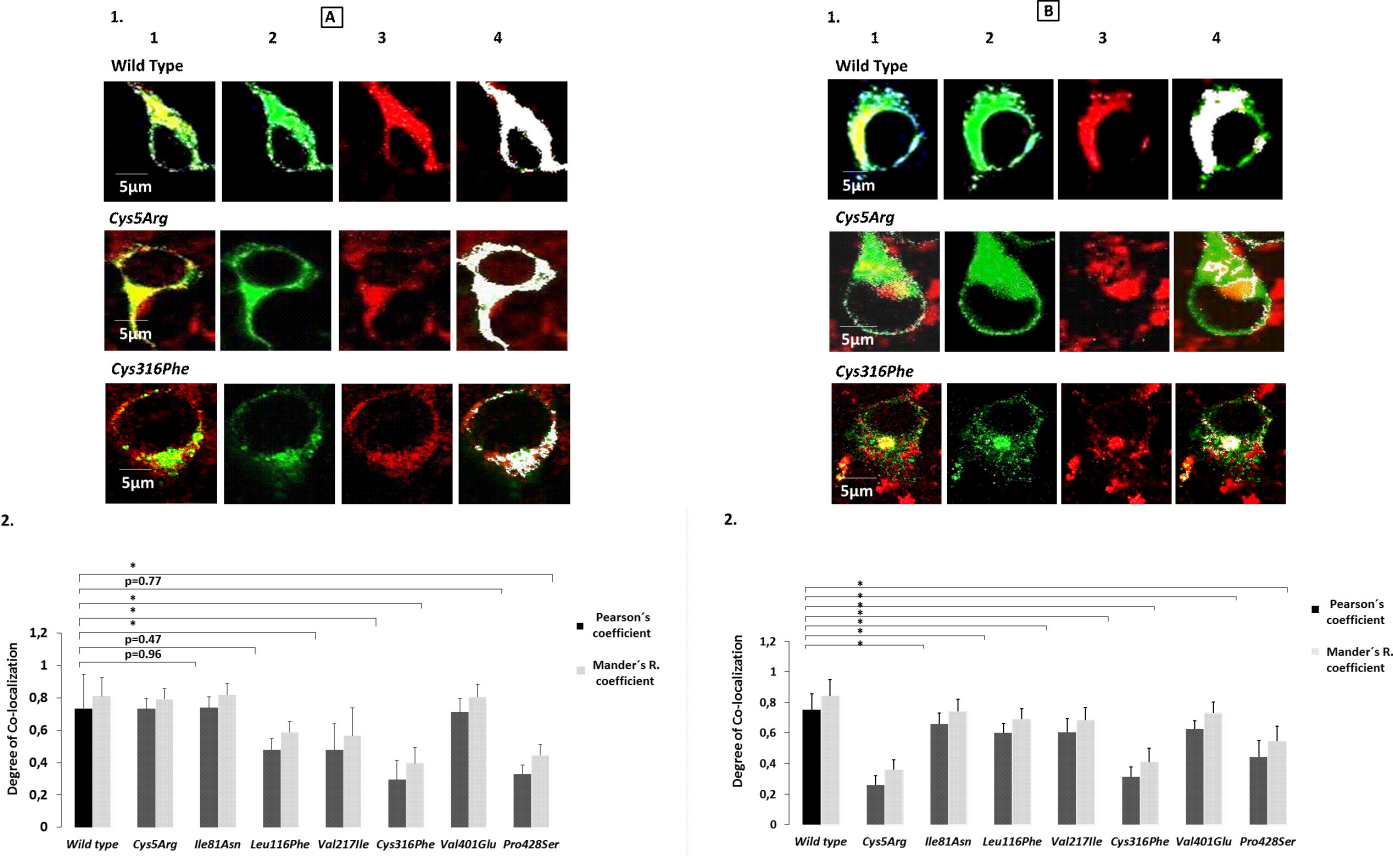
Confocal analysis and the degree of colocalization for wild-type and the reported missense mutations. A(1) Confocal images for colocalization of our wild-type and mutant variant FXIIIB subunit protein with ER. In order to avoid any confusion only the two cysteine-based mutations, *Cys5Arg* and *Cys316Phe* have been shown here. The images for all the missense mutations can be seen in Figure S3. Each representative confocal image is split into four sections: green staining representing the FXIIIB subunit protein, red staining representing the ER, yellow staining showing the colocalization of ER and FXIIIB subunit and finally white dots which also represents co-localization of ER and FXIIIB subunit. A(2) Bar graph representing comparative degree of ER colocalization with ER for the wild-type and mutant variant FXIIIB subunit protein. B(1) and B(2) is similar to A(1) and A(2), the main difference being that the red stain represents the Golgi network and the co-localization overlays shown are for the FXIIIB subunit protein with the Golgi network.

The comparative degree of colocalization expressed as Pearson and Mander′s R coefficient showed an interesting pattern with respect to one particular mutation, that is, *Cys5Arg*. The *Cys5Arg* variant showed similar levels of colocalization for ER (difference *P *= 0.96; Fig.2A) but very low levels of colocalization for Golgi (lowest amongst all mutations; difference *P* < 0.001) (Fig.2B). The degree of colocalization was also quite low in both ER and Golgi for the mutations *Cys316Phe* and *Pro428Ser*. *Ile81Asn* and *Val401Glu* showed similar levels of colocalization in ER and lower but only borderline significant levels of colocalization in Golgi indicating that their secretion paths follow similar fates as that of wild type. The *Val217Ile* mutant shows significantly lower levels of colocalization for both ER and Golgi (but not of the order of other high impact mutations like *Cys316Phe* and *Pro428Ser*) which might account for the slight difference in secreted antigen levels for this mutant when compared with the wild type.

### Proteasome inhibition by Lactacystin

The inhibition of the proteasome by Lactacystin showed a corrective effect for all mutations except *Ile81Asn* and *Val217Ile*. The mutations *Cys5Arg*, *Leu116Phe*, *Cys316Phe and Pro428Ser* show an increase in FXIIIB antigenic level more than that observed for the wild type (0.56 *μ*g/mL) post-Lactacystin treatment (Fig. S1).

### Expression and reconstitution study of the FXIIIB subunit variants with recombinant FXIIIA subunit

**Figure 3:**
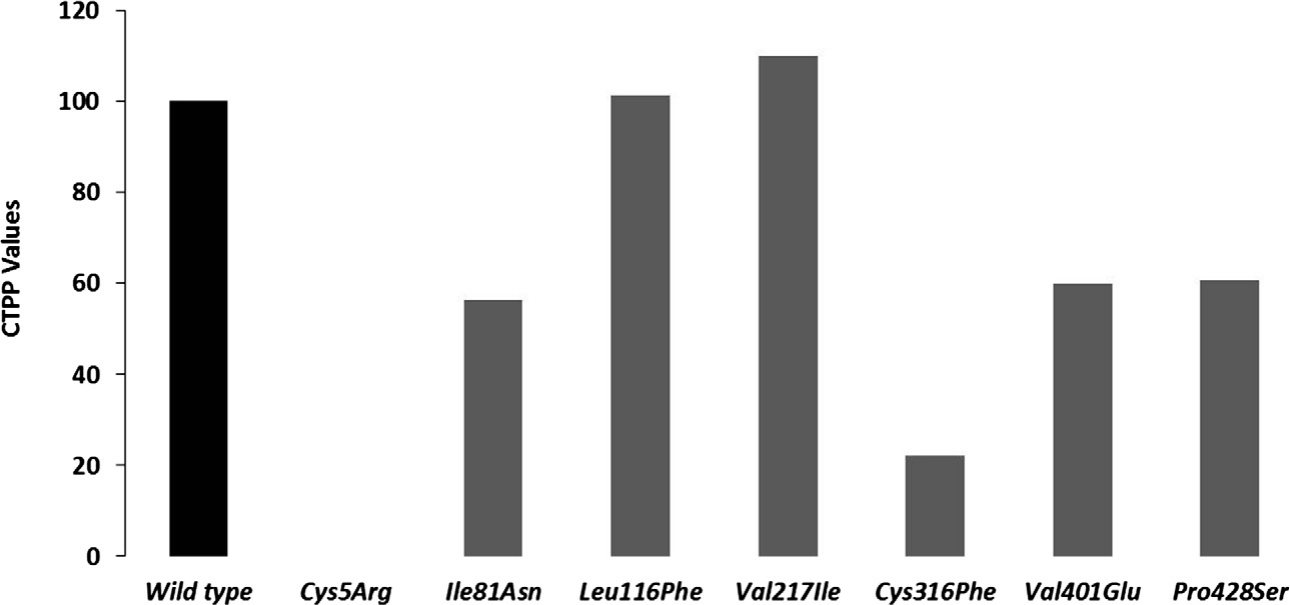
CTPP values representing FXIIIB-binding ability. This image is a comparative bar graph description of the wild type versus mutant FXIIIB subunit CTPP (comparative tetramerization potential percentage) values. The calculations for CTTP have been explained in the methods section. Since the CTTP values are a comparative representation when the wild type is considered to be 100%, therefore no error bars are shown for this figure.

Reconstitution experimentation with recombinant FXIIIA subunit showed a significant impact on binding to the FXIIIA subunit and hence on heterotetramer assembly for four FXIIIB subunit mutations (*Ile81Asn*, *Val401Glu*, *Cys316Phe,* and *Pro428Ser*) (Fig.3). The *Cys5Arg* variant showed nondetectable levels of FXIIIA_2_B_2_ antigen and therefore representative CTP or CTPP values were not calculated. The highest calculated impact on heterotetramer assembly was for the *Cys316Phe* mutation (only ∼22% of the binding ability of wild type FXIIIB). Two mutations (*Leu116Phe* and *Val217Ile*) showed CTP and CTPP values similar to wild type indicating no effect on heterotetramer assembly.

### Structural and sequence conservation analysis

**Figure 4:**
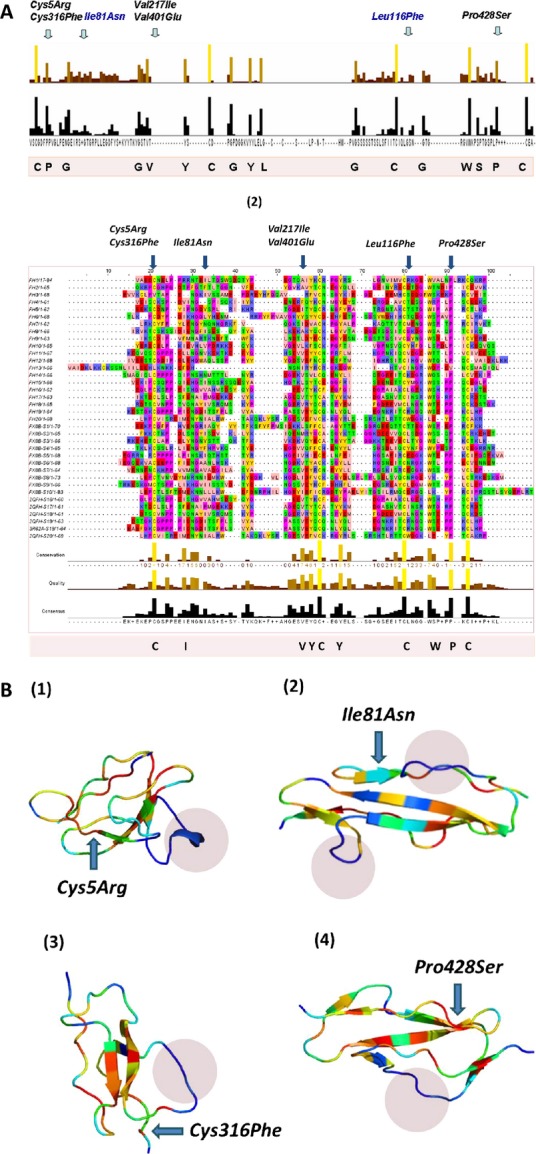
Sequence and structural conservation of sushi domains. A(1) Conservation results for the multiple sequence alignment of FXIIIB subunit sushi domains with sushi domains from 50 mammalian sushi domain-containing proteins. Owing to the large size of the actual alignment, it is not shown here. Only the conservation has been shown here with two bar graphs. The upper colored one depicts the degree of conservation and the lower bars show the consensus. Taller bars indicate higher sequence identity and also conservation. A(2) Multiple sequence alignment of FXIIIB subunit sushi domains with sushi domains of the complement factor H. The highly conserved residues are colored and also listed at the bottom of the multiple sequence alignment. The residues corresponding to each mutation have been marked at the position they occur in the alignment. (B) Structural alignment results for four sushi domains on which the mutations *Cys5Arg (S1; B1)*, *Ile81Asn (S2; B2)*, *Cys316Phe (S6; B3)* and *Pro428Ser (S7; B4)* occur. Structural conservation is depicted in a color gradient where blue represents lowest conservation level and red the highest. The variable length loops connecting the beta strands, which show the highest structural variability, are shown in blue shaded regions in each domain.

Sequence alignment with CFH sushi domains as well as with sushi domains from 50 different mammalian proteins (containing sushi domains) suggest a number of consistently conserved residues aside from the cysteines which form the backbone structural disulfide bonds of the sushi domains [Fig.4A(1) and (2)]. Interestingly most of these sequence conserved residues are hydrophobic (Proline, Tryptophan) in character suggesting a role for these residues either in binding/functional interactions or maintaining the hydrophobic core of their respective sushi domains. Structural alignment of the sushi domains differs from their sequence alignment in certain segments. We observed sequence conserved residues occurring in regions of structural variability in the FXIIIB sushi domains. This was quite frequent for the residues on the variable length loops connecting the sushi domain beta strands (Fig.4B). The two reported cysteine missense mutations occur in highly conserved structural cores of their sushi domains and show practically no variability at all (structurally as well as sequence identity based). The Pro428 residue is also highly conserved. Only in the 13th factor H sushi domain it is substituted by another residue, which turns out to be a hydrophobically similar and small Val residue [Fig.4A(2)]. The Ile81 residue occurs in a semiconserved sequence but a structurally variable region of the FXIIIB subunit S2 sushi domain. The Ile residue in the multiple sequence alignment with CFH sushi domain varies on most occasions to a hydrophobically similar Val residue [Fig.4A(2)]. Only in sushi domains 14 of CFH we observe an Asn residue similar to the reported mutation (*Ile81Asn*). However, sushi domain 14 has no designated role in protein–protein interactions for CFH protein, therefore this sushi domain might be functionally redundant (Perkins et al. 2011). In the larger multiple alignment this residue is poorly conserved but most of the variant residues are also hydrophobic in character. The Leu116 residue is located in a highly conserved sequence stretch (VQCLSDG) within which the Leu residue is the most poorly conserved residue. Interestingly, this residue varies between polar and nonpolar residues almost consistently between the various sushi domains in both the larger as well smaller (with CFH) multiple sequence alignments. The Val217 residue is a highly conserved residue and it aligns with the Val401 residue (on which another mutation *Val401Glu* has been reported) from sushi domain 7. The difference between the two mutations is that one is to a hydrophobically similar Ile residue and the other results in a negatively charged polar Glu residue.

### MD simulation analysis for FXIIIB subunit sushi domain mutations on the FXIIIB subunit sushi domain homology-based models

**Figure 5:**
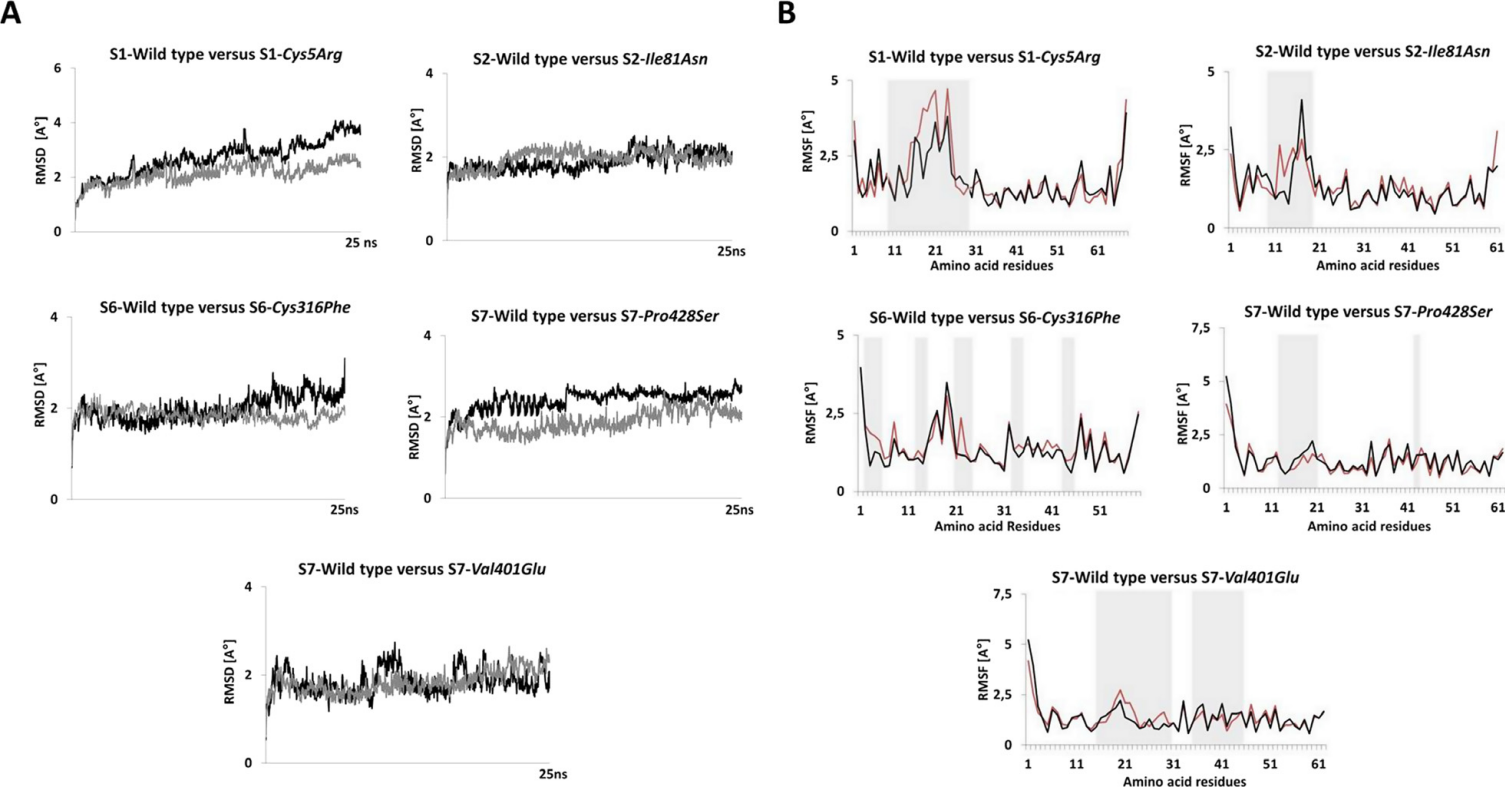
Simulation RMSD and RMSF comparisons for wild-type and mutated sushi domains. (A) Changes in RMSD during the 25 ns simulation for the mutated and wild-type sushi domains. Only the high impact mutations Cys5Arg, Cys316Phe, Pro428Ser that undergo significant changes in RMSD during simulation and one Ile81Asn mutation with no observable difference in RMSD are illustrated here. The dark black patterns represent wild-type RMSDs while the gray patterns represent the mutant RMSDs in each graph. (B) Observed RMSF per residue during simulation for the mutated and wild-type sushi domains. Only the high impact mutations Cys5Arg, Cys316Phe, Pro428Ser and two other mutations Ile81Asn and Val401Glu are shown here. The regions showing noticeable differences in RMSFs are shaded in gray. The red patterns represent the wild-type RMSFs while the black pattern represents the mutated RMSFs in each graph. The RMSD and RMSF calculation for the simulation trajectories were done on YASARA version 13.9.8. RMSD, root mean square deviation; RMSF, root mean square fluctuation.

**Figure 6:**
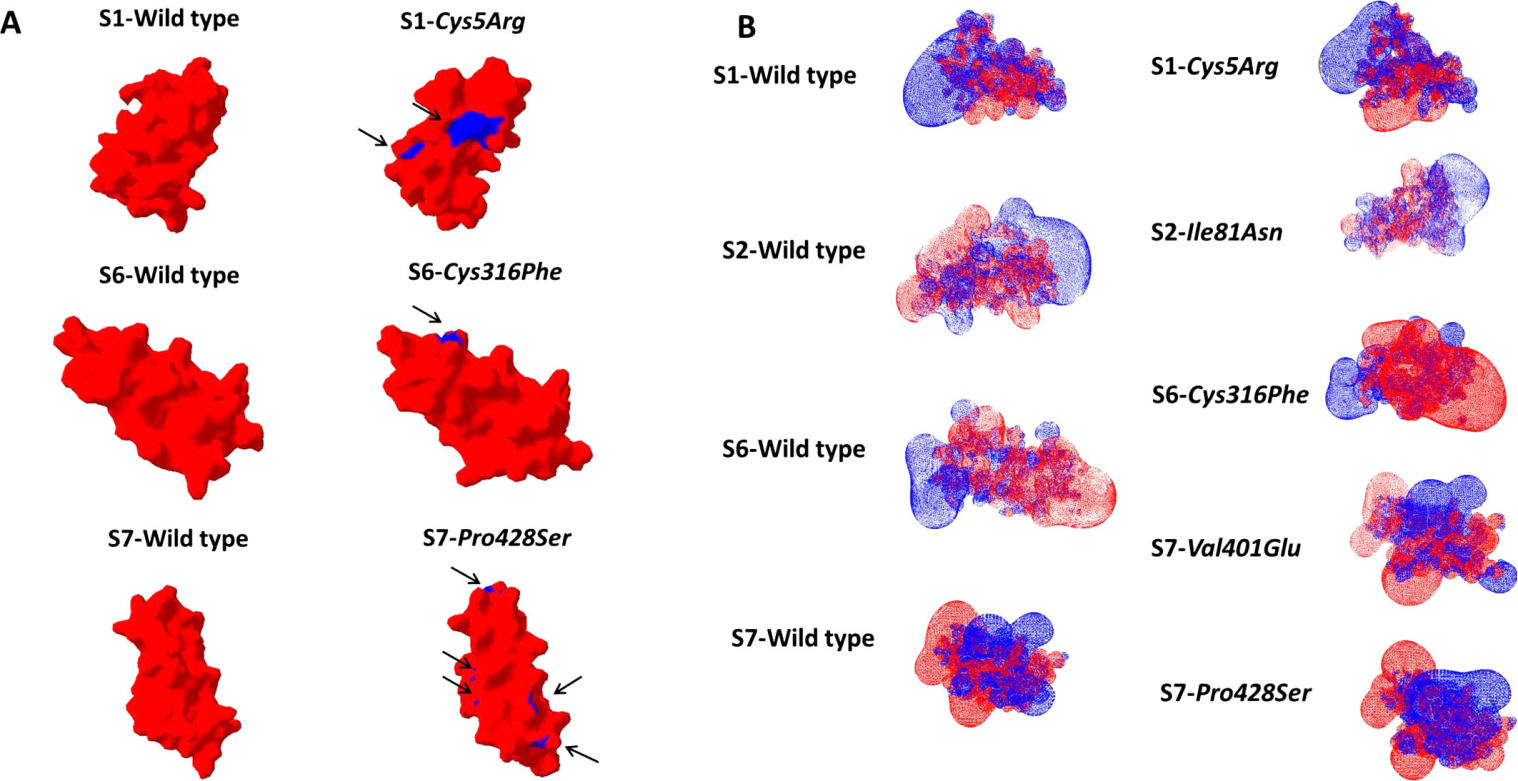
Surface electrostatic potential and Hydrophobic patch representations of Sushi domains. (A) Surface electrostatic potential map for the simulation averaged structures of wild-type sushi domains alongside the simulation-averaged structures of their corresponding mutated sushi domains. The images have been generated on SWISS-PDB viewer. The surface electrostatic potential calculations were made using the Poisson-Boltzmann method utilizing partial charges of atoms. The default dielectric constant of four for the protein and 80 for the solvent were used during calculations. An ionic strength of 50 mmol/L was used during calculations. Red color denotes negative potential while blue denotes positive potential. (B) Hydrophobic patches as determined with the SWISS-PDB viewer tool for simulation averaged structures of wild-type and mutated sushi domains. Surface representations of the sushi domains are shown. Hydrophobic patches are colored blue while the rest of the domain is colored red. The combined calculated areas of all hydrophobic patches for each sushi domain (in those detected) have been tabulated in Table S2.

**Figure 7:**
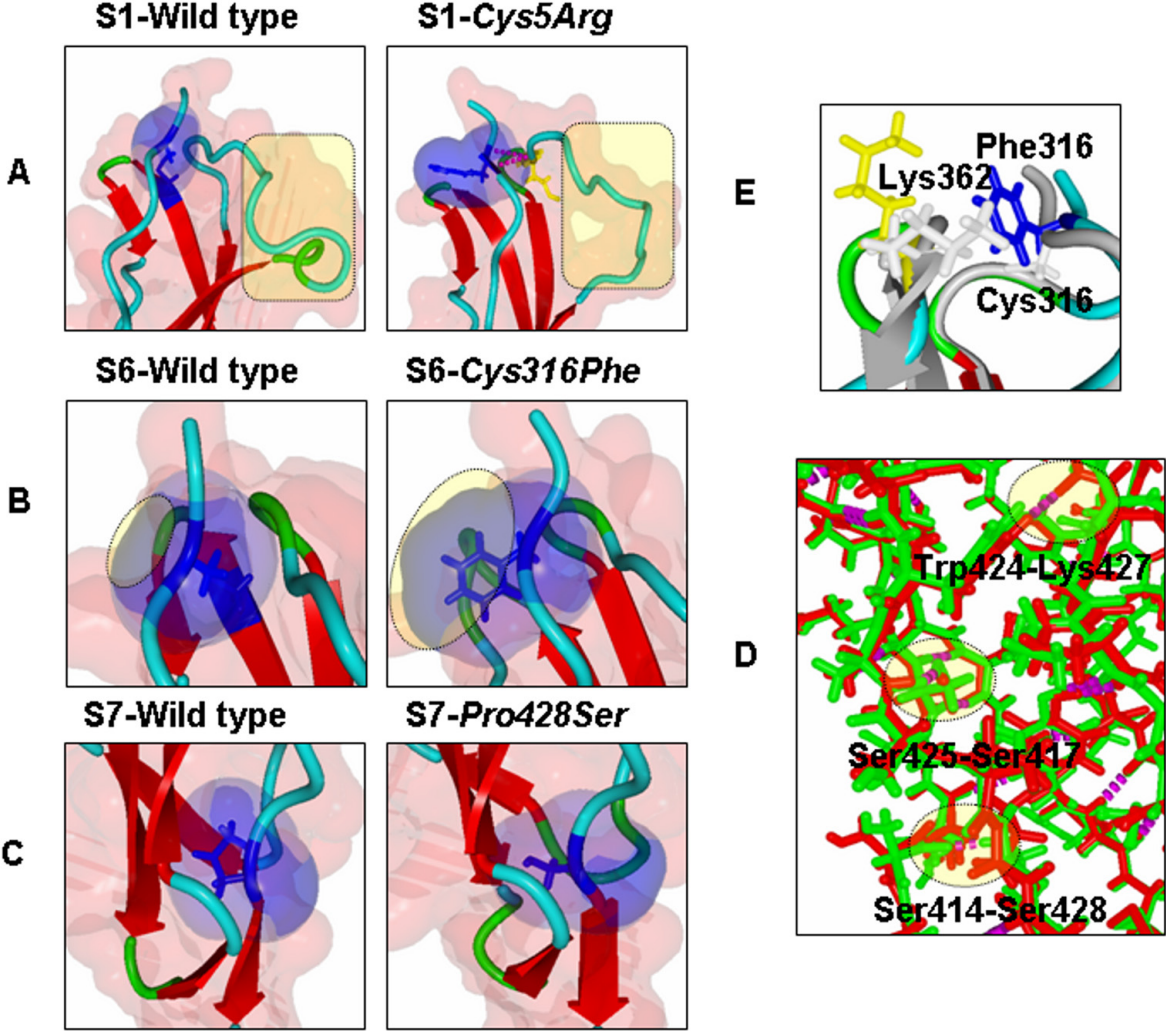
Close up views of the local molecular environment of reported missense mutations. (A–C) Depicts side-by-side local molecular environments for the mutations *Cys5Arg*, *Cys316Phe*, *Pro428Ser* with their wild-type sushi domains. The backbone is presented in ribbon format. Residue(s) of interest are depicted in stick format. Also depicted is the solvent accessible area (transparent red for the entire object and transparent blue for the residue of interest). In (A) and (B) it is quite apparent that mutation results in significant gain of accessible surface area. Also in (A) the mutated Arg residue is observed to form hydrogen bonds with Met 29. (C) Both the mutated Ser and the wild-type Proresidue look similar in terms of accessible surface areas and the secondary structure. (D) Close up view of superimposed structures of the mutated (for Cys316Phe) and wild-type S6 sushi domain. The backbones are depicted in ribbon format. The wild-type backbones as well as residues are colored gray while the backbone of the mutated sushi domain is colored with respect to its secondary structure. The mutated Phe316 residue is colored blue and the neighboring residue Lys362 is colored yellow in the mutated sushi domain. These residues are depicted as stick models in both wild-type and mutated sushi domain structures. The mutated Phe316 is surface exposed as opposed to the Cys316, which is completely buried. Also surface exposed is the Lys362 in the mutated structure while in the wild-type structure it is partially buried. (E) Superimposition of S7 wild-type sushi domain (green) and S7 mutated (for Pro428Ser) sushi domain (red) structures. Backbones are depicted as stick models. Hydrogen bonds are shown as magenta dots. The hydrogen bonds lost or gained are shaded yellow and also labeled with the residues participating in the particular bond. Structural alignments for (D) and (E) were done using the MUSTANG function embedded in YASARA.

A number of subtle as well as wide-ranging differences were observed during the analysis of the simulation trajectories/simulation averaged structures of individual mutations modeled on their respective homology modeled sushi domains. The *Cys5Arg* mutation showed deviation in structure indicated by higher RMSD values than its wild-type sushi domain 1 for the entire simulation time (Fig.5A). Simulation RMSF values showed wide differences in flexibility upon mutation on neighboring hydrophobic variable length loops (Fig.5B). Differences in cross-correlation maps were also observed especially in areas of surface exposure for the sushi domain 1 (Fig. S2). A number of hydrophobic patches were detected in the simulation averaged structure for the *Cys5Arg* mutation when compared with the wild type (Fig.6A). Differences in surface electrostatic potentials for the simulation averaged structures (Fig.6B) were observed for all mutations except for *Leu116Phe* and *Val217Ile* (not shown here). In fact the differences in calculated surface electrostatic potential between the wild-type and the mutated domain was the highest for the *Cys5Arg* mutation while the other mutations also showed modest to high differences (Table S1). The mutation to an Arg residue breaks the disulfide bond but also results in the gain of hydrogen bonds with Met29 (Fig.7B). The mutation also results in the gain of accessible surface area for the mutated Arg residue side chain instead of the completely buried wild-type Cys residue (Table S2). Two other mutations, *Cys316Phe* and *Pro428Ser* also show higher RMSD values when compared with the wild type but not of the order of *Cys5Arg* (Fig.5A). The *Cys316Phe* mutation in particular behaves close to the wild type for most of the simulation time. However, this mutation does show differences in RMSD values from that of its wild-type sushi domain when approaching the end of the stipulated simulation time, indicating a slower and lower impact mutation than the *Cys5Arg* mutation. The *Pro428Ser* mutation showed consistently higher RMSD values than the wild type during the entire simulation. The *Cys316Phe* and *Pro428Ser* mutations showed a number of differences in the cross-correlation maps (Fig. S2) indicating an impact on the correlated motion and therefore relative orientation of individual residues on the sushi domain surface. The mutation *Cys316Phe* showed one small hydrophobic patch when compared with the wild type (Fig.6A; Table S3). This hydrophobic patch is dominated by the mutated surface exposed Phenylalanine (Phe316) which lies next to a Lys362 residue which is also surface exposed in the mutated sushi domain in comparison to its wild-type domain where it is partially buried (Fig.7E). This hydrophobic patch is a likely target for the ubiquitin system for the clearance of this misfolded mutant variant by the proteasomal degradation pathway (since ubiquitin attaches itself to surface lysine residues) Mattiroli and Sixma et al. 2014. The *Pro428Ser* mutation shows conspicuous hydrophobic patches all over its sushi domain (Fig.6A). The mutated Ser428 residue seems to mimic the wild-type Proresidue since there is no observable loss or gain of surface accessible surface area or a distinct change in backbone secondary structure of the residue (Fig.7C). However, the interaction profile of the simulation averaged structure shows that this mutation leads to the loss as well as gain of several hydrogen bonds/salt bridges (Fig.7D). This might be principally responsible for altering the native fold and exposing the observed hydrophobic patches. The simulation run for the *Val401Glu* mutation does not show remarkable changes in RMSD (Fig.5A). The average RMSDs though do show small differences between the wild type and mutant (0.113 Å). The RMSF values for *Pro428Ser* and *Cys316Phe* mutations show small changes in flexibility in regions spread across the sushi domains (Fig.5B). The mutations *Ile81Asn* and *Val401Glu* also show differences in RMSF values between the mutated and the wild-type sushi domains suggesting influences on domain flexibility. The other two mutations (*Leu116Phe* and *Val217Ile*) do not show any remarkable differences in RMSD or RMSF values (data not shown here). The free energy calculations made across different servers consensually suggest a destabilizing influence for almost all mutations except for *Leu116Phe* and *Val217Ile* (Table S4). Our simulation analysis agrees best with the results obtained with the FoldX tool, Eris and Imut 2.0 server in that the two Cys mutations and one Pro mutation seem to have the highest impact on domain stability. The *Val401Glu* mutation also had high destabilizing free energy values across almost all free energy evaluation servers tested in this study.

## Discussion

The missense mutations investigated in our study have been reported in heterozygous form in patients with mild FXIII deficient phenotype. The occurrence of heterozygous mutations is a phenomenon not uncommon to sushi domain-containing proteins as was earlier observed by Goodship (2006) and Dragon-Durey et al. (2004) in CFH proteins. The heterozygous form of expression in our study conclusively demonstrates that these mutations can be classified into three major types: (1) Mutations which show influence on antigenic stability (and therefore are partially corrected for their phenotype when coexpressed with the wild type) (2) Mutations which have no influence on antigenic stability and therefore their impact is limited to interference in protein–protein interactions (i.e., dimer/heteromer assembly) and (3) Mutations which influence antigenic stability as well as binding interactions. The two cysteine mutations, *Cys316Phe* and *Cys5Arg* and the proline mutation, *Pro428Ser* clearly belong to the first as well as last group. In our earlier study we had observed differential rate/pattern of secretion for almost all FXIIIB missense mutations investigated (Biswas et al. 2013). In the current study, we have gone a step further to look for intracellular accumulation followed by possible downregulation of these mutations by the unfolded protein response. Amongst the high impact mutations, only *Cys5Arg* was observed to accumulate within the ER following which the mutant protein is cleared off by the unfolded protein response of the cell. This was clearly demonstrated since on one hand almost equivalent amount of protein was observed colocalized with the ER for the mutated protein (when compared with the wild type), almost none to nonsignificant amounts of protein was observed in the Golgi indicating an immediate response from the quality control system of the cell. The structural analysis also demonstrated that the mutation most likely results in structural destabilization (high RMSDs) and strong hydrophobic patches on the surface of the protein/domain which will elicit a strong unfolded protein response. As a result almost no protein is observed in the secreted medium for the *Cys5Arg* mutation. The influence of the unfolded protein response on the mutated protein is further demonstrated when upon inhibition of the proteasome (by Lactacystin) we observe a correction of the expression phenotype. The other two high impact mutations *Cys316Phe* and *Pro428Ser* do not seem to have an accumulative influence on the respective variant proteins as no accumulation is seen in either ER or Golgi. However, structural analysis does show the presence of hydrophobic patches as well as a destabilizing influence (high RMSDs) on their respective domains. This is further supported by the fact that while the mutated variants are observed in both Golgi and ER, their amounts are significantly reduced. This suggests that these mutations do elicit a weak unfolded protein response (in comparison to *Cys5Arg*) which leads to reduced protein secretion. The highest difference in post-Lactacystin-induced antigenic values was observed for *Pro428Ser*. While some antigen from all three of these mutations (*Cys5Arg*, *Cys316Phe*, and *Pro428Ser*) does get secreted into the medium, this variant protein is still likely to be nonfunctional owing to altered surface electrostatics which will prevent its association with the FXIIIA_2_ subunit and therefore also influence heterotetramer assembly. This is further demonstrated by the low CTP values for the *Pro428Ser* and *Cys316Phe* mutations (the *Cys5Arg* mutation was not evaluated as explained before owing to very low antigenic values). The high degree of structural and sequence conservation and the *in silico* calculated folding energy values observed for the wild-type residues corresponding to these three mutations (*Cys5Arg*, *Cys316Phe*, and *Pro428Ser*) lend further credence to the idea that these mutations primarily have a destabilizing effect on the protein. Furthermore, our confocal analysis confirms that amongst these three mutations, *Cys5Arg* is the only true accumulative/secretion-based defect resulting in ER accumulation and subsequent degradation. Interestingly, although *Cys5Arg* and *Cy316Phe* both break structural disulfide bonds their detailed expression/confocal phenotype (one results in accumulation/degradation while other shows no accumulation/lower rate of degradation) is different. This dichotomy has been observed in cysteine mutations from the sushi domains of CFH protein also. Saunders et al. (2006) tabulated that amongst eight reported cysteine substitutions which break structural disulfide bonds, six resulted in the loss of stability/incorrect folding and reduced antigen levels. The other two mutations, *p.Cys630Trp* (*c.1890T>G*) and *p.Cys1043Arg* (*c.3127T>C*) in the 11th and 17th sushi domains, respectively, showed normal antigen levels. Therefore, we hypothesize that in spite of forming structural disulfide bonds; cysteines in FXIIIB subunit (and other sushi domain-containing proteins) are likely to show strong functional diversity. The mutations *Ile81Asn* and *Val401Glu* both show near wild-type-like phenotype and slightly attenuated level phenotype, respectively, in terms of secretion/confocal phenotype. *In silico* analysis did not show any major implications for these two mutations on the stability of their respective sushi domains. What both mutations clearly show are changes in the surface electrostatic potential for their respective domains. Previous literature suggests that sushi domain interactions are primarily electrostatic mediated and that sushi domains can be classified into functional groups on the basis of their surface electrostatic properties (Soares et al. 2005). This coupled with the fact that both show reduced CTPP values (when compared with the wild type) suggests that these mutations primarily influence binding to the FXIIIA_2_ subunit and therefore FXIIIA_2_B_2_ heterotetramer assembly since native PAGE ruled out any impaired dimer formation (data not shown; although a qualitative effect cannot be ruled out). Previous literature suggests that sushi domain interactions are primarily electrostatic mediated and that sushi domains can be classified into functional groups on the basis of their surface electrostatic properties (Soares et al. 2005). The *Val401Glu* mutation might additionally have a mild destabilizing effect in addition to influencing binding to the FXIIIA subunit (as evidenced by differences in average RMSDs from the wild type, minor gain of accessible surface area, radius of gyration over the wild type and folding free energy calculations). The sushi domain 2 of the FXIIIB subunit on which the *Ile81Asn* mutation occurs has now been conclusively shown to participate in interaction with the FXIIIA subunit (Katona et al. 2014). The interface region for this sushi domain shown in this study is proximal to our reported mutation. Sushi domain 7, on which the *Val401Glu* mutation occurs has so far showed no role in FXIIIA subunit interaction or FXIIIB subunit dimerization (Souri et al. 2008). However, since the entire heterotetramer assembly is assumed to be cooperative in nature the participation of other domains has not been ruled out as yet. This mutation results in the replacement of a highly sequence and structurally conserved non polar residue with a negatively charged Glu residue which explains for the difference in the electrostatic potential surface observed for the mutated sushi domain from the wild-type sushi domain. This also explains the reduced CTPP values from our study for the *Val401Glu* mutation which further confirms its impact on heterotetramer assembly.

The remaining two mutations, *Leu116Phe* and *Val217Ile* are observed to be pathologically neutral in terms of secretion, structural and functional stability. No effect on heterotetramer assembly was observed for these two mutations. Low degree of sequence and structural conservation further supports the neutral status of these two mutations. Minor influences on the rate of biosynthesis still cannot be ruled out for these two variant within the current experimental set up. Even in the event of a minor influence, these variants are most likely candidate functional polymorphisms rather than true causative mutations.

We have therefore using a host of functional, structural/computational analysis successfully determined the underlying molecular mechanisms for the causality of seven FXIIIB subunit variants which we had earlier reported in heterozygous form from patients with mild FXIII deficiency. We found that three of these mutations (*Cys5Arg*, *Cys316Phe*, and *Pro428Ser*) cause antigenic instability as well as reduced binding to FXIIIA subunit, two (*Ile81Asn* and *Val401Glu*) cause impaired FXIIIA subunit binding while maintaining antigenic stability. Only one mutation in the entire lot (*Cys5Arg*) showed a true accumulative/secretion-based defect while two (*Leu116Phe* and *Val217Ile*) others seem to have neutral status.

## Supporting Information

**Figure S1.** Proteasomal inhibition by lactacystin. The bar graphs in this image represent the difference in FXIIIB antigen values with and without lactacystin treatment (Proteasome inhibition). The wild type is represented by dark black bar while all the other mutations are represented by dark gray bars. A dotted black line marks the difference in antigenic level (i.e., for the wild type) above which any observation would be considered an effect on proteasome due to the mutation. **Figure S2.** DCCM map for simulation trajectories. This image depicts the DCCM (dynamic cross-correlation matrices) map of each mutation’s simulation trajectory compared with its wild-type sushi domain simulation trajectory. The yellow regions show positive correlation (proportional to its intensity) and the blue regions shows negative correlation. **Figure S3.** Confocal microscopy images for all variants. This image shows the co-localization pattern for all missense mutations with ER and Golgi in comparison with the wild type. The image coding is the same as in the main Figure 2. **Table S1.** Surface electrostatic potential differences between the mutant and wild-type sushi domains. **Table S2.** Accessible surface area and radius of gyration for simulation averaged wild-type and mutant structures. **Table S3.** Exposed hydrophobic patches for each mutation. **Table S4.** Free energy changes calculated for each mutation across various protein stability prediction servers.

## References

[b1] Adler J, Parmryd I (2010). Quantifying colocalization by correlation: the Pearson correlation coefficient is superior to the Mander’s overlap coefficient. Cytometry A.

[b2] Biswas A, Ivaskevicius V, Seitz R, Thomas A, Oldenburg J (2011). An update of the mutation profile of Factor 13 A and B genes. Blood Rev.

[b3] Biswas A, Thomas A, Bevans CG, Ivaskevicius V, Oldenburg J (2013). In vitro secretion deficits are common among human coagulation factor XIII subunit B missense mutants: correlations with patient phenotypes and molecular models. Hum. Mutat.

[b4] Biswas A, Ivaskevicius V, Thomas A, Oldenburg J (2014a). Coagulation factor XIII deficiency. Diagnosis, prevalence and management of inherited and acquired forms. Hamostaseologie.

[b5] Biswas A, Ivaskevicius V, Thomas A, Varvenne M, Brand B, Rott H (2014b). Eight novel F13A1 gene missense mutations in patients with mild FXIII deficiency: in silico analysis suggests changes in FXIII-A subunit structure/function. Ann. Hematol.

[b6] Capriotti E, Fariselli P, Casadio R (2005). I-Mutant2.0: predicting stability changes upon mutation from the protein sequence or structure. Nucleic Acids Res.

[b7] Dragon-Durey MA, Fremeaux-Bacchi V, Loirat C, Blouin J, Niaudet P, Deschenes G (2004). Heterozygous and homozygous factor h deficiencies associated with hemolytic uremic syndrome or membranoproliferative glomerulonephritis: report and genetic analysis of 16 cases. J. Am. Soc. Nephrol.

[b8] Gaboriaud C, Rossi V, Bally I, Arlaud GJ, Fontecilla-Camps JC (2000). Crystal structure of the catalytic domain of human complement c1s: a serine protease with a handle. EMBO J.

[b9] Goodship TH (2006). Factor H genotype-phenotype correlations: lessons from aHUS, MPGN II, and AMD. Kidney Int.

[b10] Guex N, Peitsch MC, Schwede T (2009). Automated comparative protein structure modeling with SWISS-MODEL and Swiss-PdbViewer: a historical perspective. Electrophoresis.

[b11] Ichinose A, McMullen BA, Fujikawa K, Davie EW (1986). Amino acid sequence of the b subunit of human factor XIII, a protein composed of ten repetitive segments. Biochemistry.

[b12] Ivaskevicius V, Biswas A, Bevans C, Schroeder V, Kohler HP, Rott H (2010a). Identification of eight novel coagulation factor XIII subunit A mutations: implied consequences for structure and function. Haematologica.

[b13] Ivaskevicius V, Biswas A, Loreth R, Schroeder V, Ohlenforst S, Rott H (2010b). Mutations affecting disulphide bonds contribute to a fairly common prevalence of F13B gene defects: results of a genetic study in 14 families with factor XIII B deficiency. Haemophilia.

[b14] Katoh K, Standley DM (2013). MAFFT multiple sequence alignment software version 7: improvements in performance and usability. Mol. Biol. Evol.

[b15] Katona E, Penzes K, Csapo A, Fazakas F, Udvardy ML, Bagoly Z (2014). Interaction of factor XIII subunits. Blood.

[b16] Konc J, Janezic D (2010a). ProBiS: a web server for detection of structurally similar protein binding sites. Nucleic Acids Res.

[b17] Konc J, Janezic D (2010b). ProBiS algorithm for detection of structurally similar protein binding sites by local structural alignment. Bioinformatics.

[b18] Kopp A, Hebecker M, Svobodova E, Jozsi M (2012). Factor h: a complement regulator in health and disease, and a mediator of cellular interactions. Biomolecules.

[b20] Krieger E, Koraimann G, Vriend G (2002). Increasing the precision of comparative models with YASARA NOVA–a self-parameterizing force field. Proteins.

[b21] Krivov GG, Shapovalov MV, Dunbrack RL (2009). Improved prediction of protein side-chain conformations with SCWRL4. Proteins.

[b22] Lorand L, Losowsky MS, Miloszewski KJ (1980). Human factor XIII: fibrin-stabilizing factor. Prog. Hemost. Thromb.

[b23] Mattiroli F, Sixma TK (2014). Lysine-targeting specificity in ubiquitin and ubiquitin-like modification pathways. Nat. Struct. Mol. Biol.

[b24] Perkins SJ, Nan R, Li K, Khan S, Miller A (2012). Complement factor H-ligand interactions: self-association, multivalency and dissociation constants. Immunobiology.

[b25] Pires DE, Ascher DB, Blundell TL (2014a). mCSM: predicting the effects of mutations in proteins using graph-based signatures. Bioinformatics.

[b26] Pires DE, Ascher DB, Blundell TL (2014b). DUET: a server for predicting effects of mutations on protein stability using an integrated computational approach. Nucleic Acids Res.

[b27] Radek JT, Jeong JM, Wilson J, Lorand L (1993). Association of the A subunits of recombinant placental factor XIII with the native carrier B subunits from human plasma. Biochemistry.

[b28] Richter S, Wenzel A, Stein M, Gabdoulline RR, Wade RC (2008). webPIPSA: a web server for the comparison of protein interaction properties. Nucleic Acids Res.

[b29] Saunders RE, Goodship TH, Zipfel PF, Perkins SJ (2006). An interactive web database of factor H-associated hemolytic uremic syndrome mutations: insights into the structural consequences of disease-associated mutations. Hum. Mutat.

[b30] Schneider CA, Rasband WS, Eliceiri KW (2012). NIH Image to ImageJ: 25 years of image analysis. Nat. Methods.

[b31] Soares DC, Gerloff DL, Syme NR, Coulson AF, Parkinson J, Barlow PN (2005). Large-scale modelling as a route to multiple surface comparisons of the CCP module family. Protein Eng. Des. Sel.

[b32] Souri M, Kaetsu H, Ichinose A (2008). Sushi domains in the B subunit of factor XIII responsible for oligomer assembly. Biochemistry.

[b33] Van Durme J, Delgado J, Stricher F, Serrano L, Schymkowitz J, Rousseau F (2011). A graphical interface for the FoldX forcefield. Bioinformatics.

[b34] Waterhouse AM, Procter JB, Martin DM, Clamp M, Barton GJ (2009). Jalview Version 2–a multiple sequence alignment editor and analysis workbench. Bioinformatics.

[b35] Worth CL, Preissner R, Blundell TL (2011). SDM–a server for predicting effects of mutations on protein stability and malfunction. Nucleic Acids Res.

[b36] Yin S, Ding F, Dokholyan NV (2007). Eris: an automated estimator of protein stability. Nat. Methods.

