# Supplementary material for: Structural and functional influences of coagulation factor XIII subunit B heterozygous missense mutants
Source: Mol Genet Genomic Med. 2015 Apr 10;3(4):258–71. doi: 10.1002/mgg3.138 (PMC4521963; doi:10.1002/mgg3.138)
Supplement: Supplementary file 1 [file mgg30003-0258-sd1.doc]

*Human Mutation*

**Supplementary files**

**Structural and functional influences of coagulation factor XIII subunit B heterozygous missense mutants**

Anne Thomas^1†^, Arijit Biswas^1†^*, Vytautas Ivaskevicius^1^, Johannes Oldenburg^1*^

^1^Institute of Experimental Haematology and Transfusion Medicine, University Clinic Bonn, 53105 Bonn, Germany;

Running head title: Molecular defects underlying FXIIIB missense mutations

^†^These authors contributed equally to this work.

*Correspondence to:

Arijit Biswas, Institute of Experimental Haematology and Transfusion Medicine,

University Clinic Bonn, Sigmund Freud Str. 25, 53127 Bonn, Germany.

Email: arijit.biswas@ukb.uni-bonn.de

phone: +49 228 287 19428

fax: +49 228 287 14320

or

Johannes Oldenburg, Institute of Experimental Haematology and Transfusion Medicine,

University Clinic Bonn, Sigmund Freud Str. 25, 53105 Bonn, Germany.

Email: johannes.oldenburg@ukb.uni-bonn.de

phone: +49 228 287 15175

fax: +49 228 287 14783

**Figure S1. Proteasomal inhibition by lactacystin.** The bar graphs in this image represent the difference in FXIIIB antigen values with and without lactacystin treatment (Proteasome inhibition). The wild type is represented by dark black bar while all the other mutations are represented by dark grey bars. A dotted black line marks the difference in antigenic level (i.e. for the wild type) above which any observation would be considered an effect on proteasome due to the mutation.


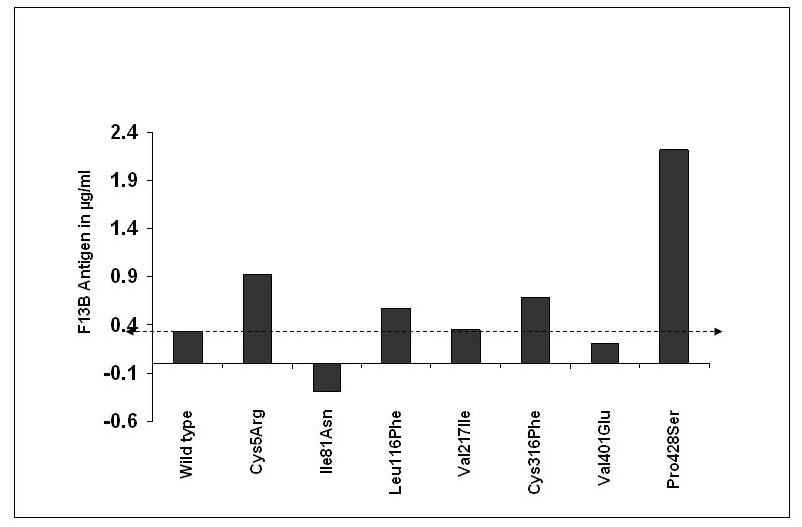


**Figure S2. DCCM map for simulation trajectories.** This image depicts the DCCM (dynamic cross correlation matrices) map of each mutation´s simulation trajectory compared with its wild type sushi domain simulation trajectory. The yellow regions show positive correlation (proportional to its intensity) and the blue regions shows negative correlation.

**
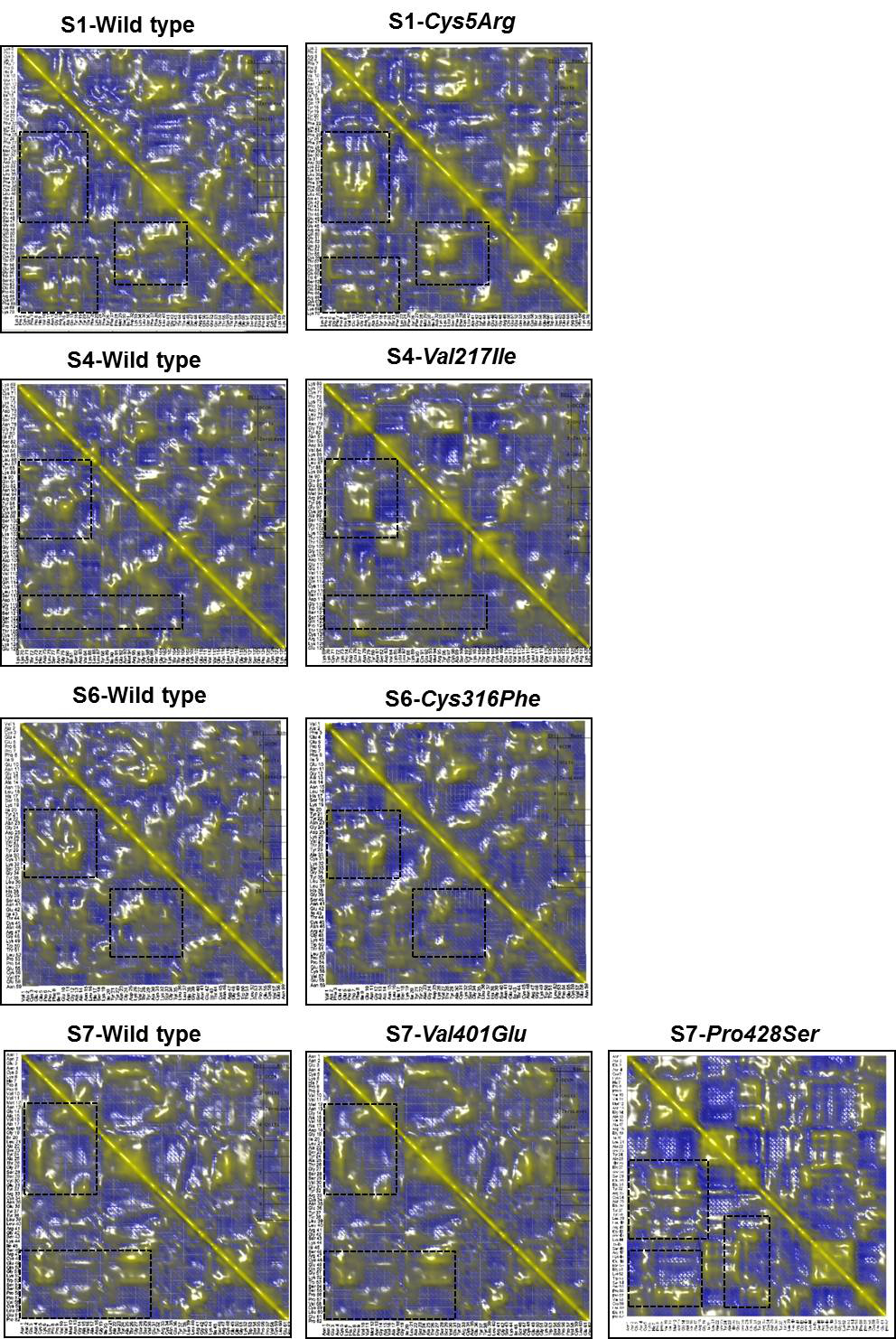
**

**Figure S3. Confocal microscopy images for all variants. This image shows the co-localization pattern for all missense mutations with ER and Golgi in comparison with the wild type. The image coding is the same as in the main Figure 2.**

**
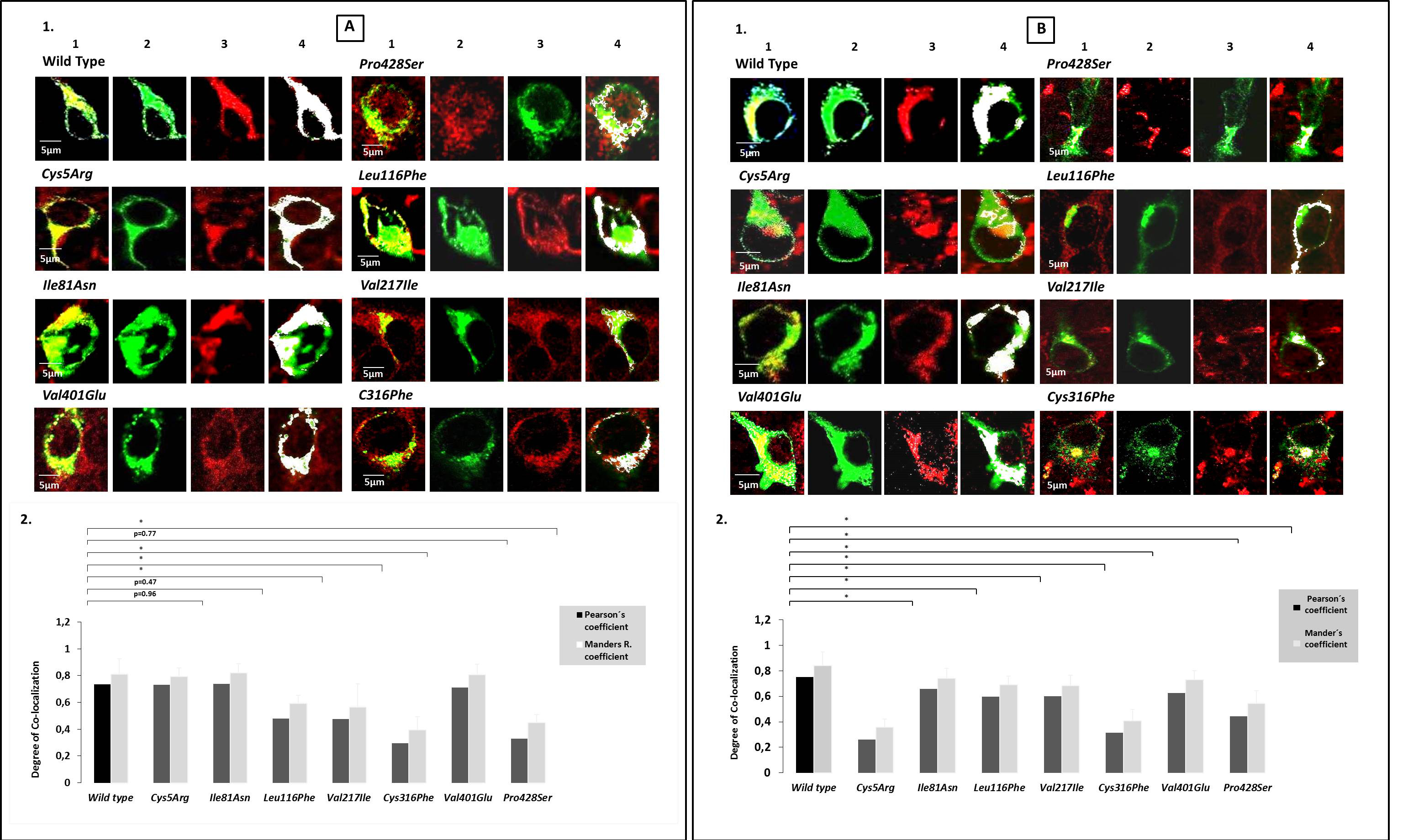
**

**Table S1. Surface electrostatic potential differences between the mutant and wild type sushi domains.**

Distance/difference values range from 0-2. Higher values represent larger differences/distances.

| **Mutation vs Wild type** | **Surface electrostatic potential difference/distance** |
| --- | --- |
| Cys5Arg vs S1 | 0.61482 |
| Ile81Asn vs S2 | 0.36878 |
| Leu116Phe vs S2 | 0.10232 |
| Val217Ile vs S4 | 0.11372 |
| Cys316Phe vs S6 | 0.24495 |
| Pro428Ser vs S7 | 0.48785 |
| Val401Glu vs S7 | 0.38471 |

**Table S2. Accessible surface area and radius of gyration for simulation averaged wild type and mutant structures.**

Abbreviations: WT: Wild type, ROG: Radius of gyration, MT: Mutant type, ASA: Accessible surface area.

| **WT** | **ASA(Å^2^)** | **ROG(Å)** | **MT** | **ASA(Å^2^)** | **ROG(Å)** | **MT** | **ASA(Å^2^)** | **ROG(Å)** |
| --- | --- | --- | --- | --- | --- | --- | --- | --- |
| S1 | 4823.9 | 12.421 | Cys5Arg | 5172.92 | 12.545 |  |  |  |
| S2 | 3959.05 | 11.852 | Ile81Asn | 4007.93 | 12.075 | Leu116Phe | 4008 | 11.932 |
| S4 | 4155.98 | 12.089 | Val217Ile | 4162.06 | 12.124 |  |  |  |
| S6 | 3975.92 | 12.048 | Cys316Phe | 4164.9 | 12.263 |  |  |  |
| S7 | 3983.23 | 12.693 | Val401Glu | 4135.44 | 13.104 | Pro428Ser | 4427.03 | 12.157 |

**Table S3. Exposed hydrophobic patches for each mutation.**

| **Mutation** | **Surface area(Å^2^)** |
| --- | --- |
| Cys5Arg | 175 |
| Ile81Asn | None |
| Leu116Phe | None |
| Val217Ile | None |
| Cys316Phe | 31 |
| Val401Glu | None |
| Pro428Ser | 184 |

**Table S4. Free Energy changes calculated for each mutation across various protein stability prediction servers.** Free energy values (ΔΔG) expressed as Kcal/mol. Interpretation for the FOLDX and ERIS server: ΔΔG>0, mutation is destabilizing; for other servers: ΔΔG<0 mutation is destabilizing

| **Mutation** | **SDM** | **FoldX** | **mCSM** | **DUET** | **Imutant 2.0** | **Eris** |
| --- | --- | --- | --- | --- | --- | --- |
| Cys5Arg | -0.88 | 15.61 | -1.07 | -0.769 | -1.95 | 3.32 |
| Ile81Asn | -3.54 | 1.78 | -1.17 | -1.165 | 0.92 | 3.92 |
| Leu116Phe | 0.19 | 0.56 | -1.332 | -1.442 | 1.75 | 2.59 |
| Val217Ile | 0.17 | 0.72 | -0.486 | -0.159 | 0.7 | 1.06 |
| Cys316Phe | -0.56 | 9.12 | -0.951 | -1.139 | -2.14 | >10 |
| Val401Glu | -3.04 | 1.07 | -1.652 | -1.816 | -1.02 | >10 |
| Pro428Ser | -1.92 | 12.41 | -1.749 | -1.833 | -3.22 | 5.17 |
